# The Methionine 549 and Leucine 552 Residues of Friedelin Synthase from *Maytenus ilicifolia* Are Important for Substrate Binding Specificity

**DOI:** 10.3390/molecules26226806

**Published:** 2021-11-11

**Authors:** Bruna F. Mazzeu, Tatiana M. Souza-Moreira, Andrew A. Oliveira, Melissa Remlinger, Lidiane G. Felippe, Sandro R. Valentini, Rafael V. C. Guido, Cleslei F. Zanelli, Maysa Furlan

**Affiliations:** 1 Instituto de Química, Universidade Estadual Paulista-UNESP, CP 355, Araraquara 14800-900, SP, Brazil; bruna.mazzeu@gmail.com (B.F.M.); souzatm@gmail.com (T.M.S.-M.); melissa.remlinger@yahoo.com.br (M.R.); lidiane_iq@yahoo.com.br (L.G.F.); 2 Centro de Pesquisa e Inovação em Biodiversidade e Fármacos, Instituto de Física de São Carlos, Universidade de São Paulo, São Carlos 13563-120, SP, Brazil; albert.andrew@gmail.com (A.A.O.); rvcguido@ifsc.usp.br (R.V.C.G.); 3 Faculdade de Ciências Farmacêuticas, Universidade Estadual Paulista-UNESP, Rod. Araraquara-Jaú km 1, Araraquara 14800-903, SP, Brazil; sandro.valentini@unesp.br (S.R.V.); cleslei.zanelli@unesp.br (C.F.Z.)

**Keywords:** *Maytenus ilicifolia*, Celastraceae, *Saccharomyces cerevisiae*, friedelin synthase, site-directed mutation, Met549Ser, Leu552Phe

## Abstract

Friedelin, a pentacyclic triterpene found in the leaves of the Celastraceae species, demonstrates numerous biological activities and is a precursor of quinonemethide triterpenes, which are promising antitumoral agents. Friedelin is biosynthesized from the cyclization of 2,3-oxidosqualene, involving a series of rearrangements to form a ketone by deprotonation of the hydroxylated intermediate, without the aid of an oxidoreductase enzyme. Mutagenesis studies among oxidosqualene cyclases (OSCs) have demonstrated the influence of amino acid residues on rearrangements during substrate cyclization: loss of catalytic activity, stabilization, rearrangement control or specificity changing. In the present study, friedelin synthase from *Maytenus ilicifolia* (Celastraceae) was expressed heterologously in *Saccharomyces cerevisiae*. Site-directed mutagenesis studies were performed by replacing phenylalanine with tryptophan at position 473 (Phe473Trp), methionine with serine at position 549 (Met549Ser) and leucine with phenylalanine at position 552 (Leu552Phe). Mutation Phe473Trp led to a total loss of function; mutants Met549Ser and Leu552Phe interfered with the enzyme specificity leading to enhanced friedelin production, in addition to α-amyrin and β-amyrin. Hence, these data showed that methionine 549 and leucine 552 are important residues for the function of this synthase.

## 1. Introduction

**Figure 1:**
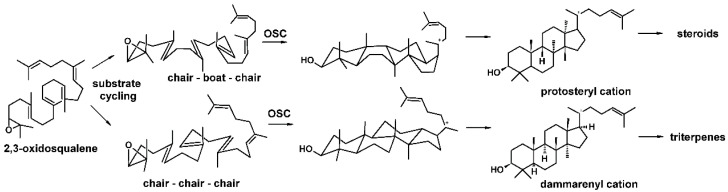
Schematic biosynthesis of the key triterpene and sterol intermediates from 2,3-oxidosqualene cyclization [8,9].

Triterpene is a class of compounds in which the carbon skeleton bearing 30 carbons originated from isoprene units [1]. They play an important structural and hormonal role in plants as well as exhibiting defensive properties [2,3]. In prokaryotes, their biosynthesis occurs directly by squalene cyclization resulting in hopanoid triterpenes [4]. Meanwhile, in eukaryotes, squalene is first converted into 2,3-oxidosqualene, followed by a cyclization reaction, yielding cycloartenol (precursor of phytosterols), pentacyclic triterpenes in plants or lanosterol in fungi and mammals, resulting in ergosterol and cholesterol, respectively [4,5]. The cyclization reaction of 2,3-oxidosqualene is catalyzed by enzymes known as oxidosqualene cyclases (OSCs) [6]. In general, OSCs are product specific, but in plants, they compete for the same substrate to produce steroids and pentacyclic triterpenes. As a result, these enzymes play a pivotal role at the border between primary and secondary metabolism [7]. The cyclization reaction of OSC requires activation by epoxide protonation, followed by a concerted intramolecular attack of double bonds, leading to a chair–boat–chair conformation, yielding the protosteryl cation, which is the key intermediate to produce steroidal skeleton, or to chair–chair–chair conformation, resulting in dammarenyl cation, the source of triterpenes [8,9] (Figure 1).

**Figure 2:**
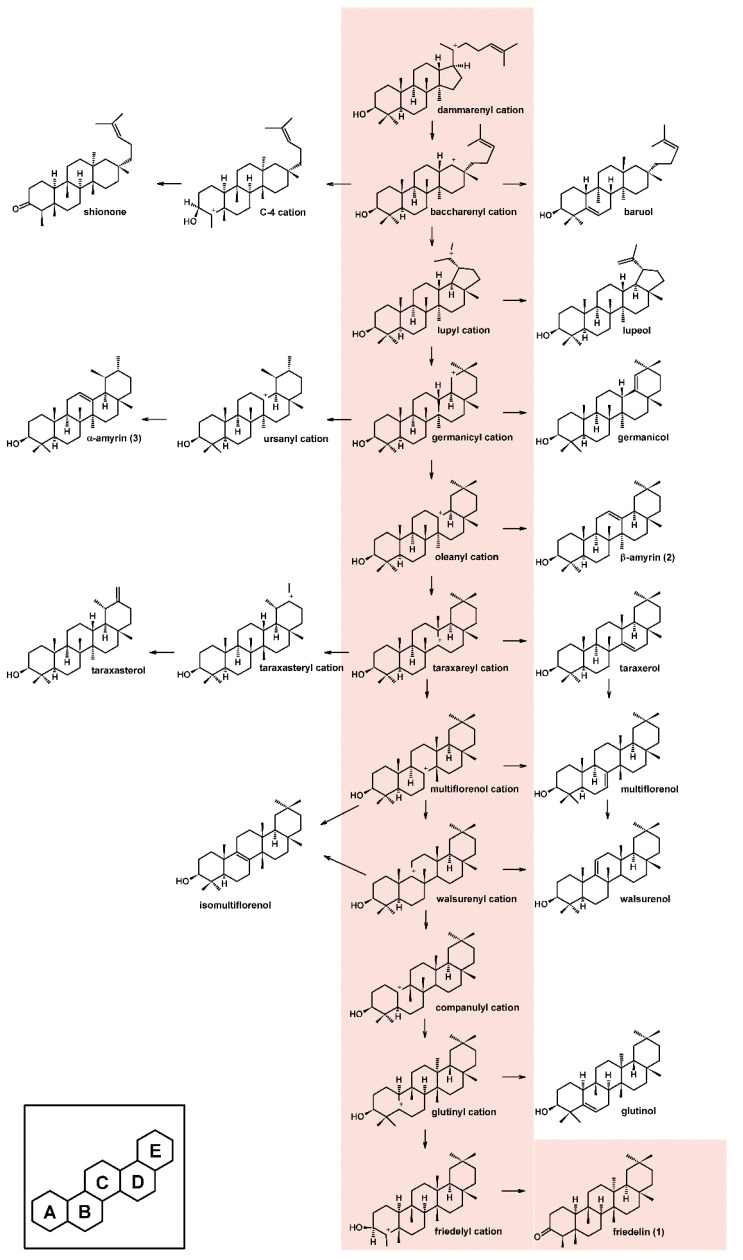
Cation rearrangement involved in the production of friedelin and other triterpenes [10]. Pink highlighted area represents the route of production of friedelin.

The oxidosqualene cyclase enzymes present in the cyclization reaction in the dammarenyl cation pathway are responsible for producing many kinds of pentacyclic triterpenes in addition to the tetracyclic shionone [10]. Friedelin synthase produces friedelin, which involves the highest number of rearrangements in the triterpene biosynthetic pathways (Figure 2). It is hypothesized that an aspartate residue starts the carbocation reaction. Then, it promotes the hydroxylation of an intermediate, which is deprotonated by the same residue yielding a ketone. All steps described until ketone formation are conducted without the help of an oxidoreductase enzyme [6,7].

Friedelin, a pentacyclic triterpene found in the Celastraceae, Euphorbiaceae, Flacourtiaceae and Clusiaceae families, has demonstrated numerous biological activities and it is a precursor of quinonemethide triterpenes in Celastraceae species, which has promising antitumoral activity [11,12]. Friedelin accumulates in the leaves of *Maytenus ilicifolia* (Celastraceae), known in Brazil as “Espinheira Santa”, which is traditionally used for stomach disorders [13]. Friedelin also shows great medicinal potential as an anti-inflammatory, analgesic, anti-pyretic, gastroprotective and antibiotic against the nonpathogenic bacteria *Mycobacterium madagascariense* and *Mycobacterium indicus pranii*, the bacterium responsible for bovine and human tuberculosis [14,15,16,17]. Friedelin is also effective in lowering the lipid levels in hyperlipidemic rats [18].

Therefore, studies of friedelin synthase activity are essential to understand its ability to promote the highest number of rearrangements towards friedelin production, its specificity, and its efficacy; these results will determine the key enzyme for heterologous expression in a *Saccharomyces cerevisiae* as a model for friedelin and quinonemethide triterpenes production through synthetic biology [19].

Several mutagenic studies of different OSCs have been performed to assess the importance of amino acid residues involved in their catalytic activities and specificities. It was shown that small changes in the structure of the active site might alter the specificity and product yield of these enzymes. This was observed in the mutants Phe728His of β-amyrin synthase from *Euphorbia tirucalli* (EtAS), which starts to produce germanicol as the main product [20], and Ile481Val of cycloartenol synthase, which loses its specificity and produces a mixture of cycloartenol, lanosterol and parkeol [21]. In the Ser728Phe mutant of β-amyrin synthase from *Avena strigosa* (SAD1), the change from serine to phenylalanine led to the stabilization of the dammarenyl cation, generating the tetracyclic triterpene dammarane [22]. In β-amyrin synthase from *Panax ginseng*, the mutation Trp259Leu led to the production of lupeol and β-amyrin; however, lupeol was the major product [23]. In contrast, the mutation Leu256Trp in lupeol synthase produced predominantly β-amyrin, showing that this tryptophan residue is crucial for β-amyrin production, whereas this leucine residue may play an essential role in lupeol biosynthesis.

Regarding all known friedelin synthases, a unique leucine residue was observed at two positions in the catalytic site. Site-directed mutagenesis studies with friedelin synthase from *M. ilicifolia* (*Mi*FRS) and *Tripterygium wilfordii* (TwOSC1) suggested the influence of leucine on the specificity of the enzymes that produced friedelin and β-amyrin or only β-amyrin depending on the residue changed [24]. On the other hand, another mutant of friedelin synthase, TwOSC1, with threonine 502 changed to glutamic acid, was able to increase the heterologous production of friedelin instead of changing its specificity, showing the potential impact of mutagenesis studies in understanding and improving friedelin production. Therefore, the goal of this paper was to evaluate the role of amino acid residues of friedelin synthase from *M. ilicifolia* involved in enzyme specificity and efficiency.

## 2. Results

### 2.1. Determination of Friedelin Synthase Mutants

To select potential amino acid residues involved in the activity of friedelin synthase from *M. ilicifolia* (*Mi*FRS, GenBank accession number MK526901, codon optimized for expression in *Saccharomyces cerevisiae*), a multiple global alignment of the primary sequences of oxidosqualene cyclase and *Mi*FRS was performed. The alignment of the selected residues and GenBank accession number of the sequences are presented in Supplementary Figure S1 and Table S1. *Mi*FRS showed high identity with various sequences of β-amyrin synthase (65–74%), and it presented approximately 65% identity with the nonspecific friedelin synthase of *Kalanchoe daigremontiana* (*Kd*FRS) [19], 74% to the friedelin synthase of *Populus davidiana* (PdFRS), and 87% and 93% to the nonspecific friedelin synthases TwOSC1 and TwOSC3 from *Tripterygium wilfordii*, respectively. The amino acids evaluated in the present study were conserved among the friedelin synthases with high identity (except for *Kd*FRS).

**Figure 3:**
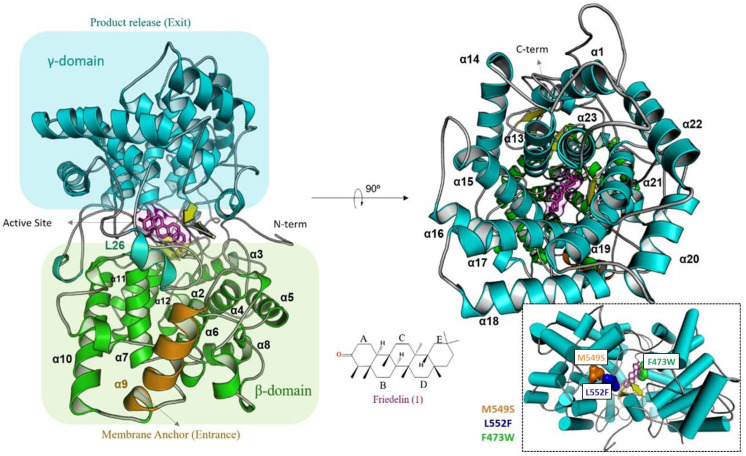
In silico model of the enzyme friedelin synthase from *M. ilicifolia* (*Mi*FRS). Secondary structure representation shows the α/α domains known as βγ-protein. The α9 helix approaches the lipid bilayer which sources the substrate. On the other side, the γ-domain processes and release the friedelin product. Horizontally to the N-terminal is organized the active site, created in interface between γ and β domains. Specifically, flexible loops and three β-hairpins give motion and polarity for the substrate production. Mutations made are located in loop regions as follows: L23 (Phe473Trp), L26 (Leu552Phe) and (Met549Ser). Native amino acids overview is highlighted by surfaces in colors on their positions inside the folding.

The molecular modeling of the globular monomeric friedelin synthase from *M. ilicifolia* was previously described [19]. It is formed by 23 α-helices, giving the topology presented in Figure 3, and is subdivided into two α/α domains known as βγ, which are similar to domains found in G βγ complex proteins. This monotopic membrane protein is connected to the bilayer structure through an α-helices (α9), which is where the substrate enters. After rearranging the internal α/α barrel in the β-domain, the solvent channel formed throughout the 65 Å diameter structure directs the substrate to the active site localized between domains. Each α/α domain is connected by type I β-hairpins, small α-turns and extended loops, considering the N-/C-terminal region of the secondary structures that surrounds the center of the subunit interface and close the reaction site. Second, the β-domain comprises an α/α barrel responsible for starting the cyclization reaction after protonation of the prefolded friedelyl cation from Asp484 located in the conserved DCTAE (Asp-Cys-Thr-Ala-Glu) motif. The region of the catalytic site comprises a triad of β-hairpins, and the flexibility of those loops enables the rearrangement of the substrate inside the catalytic site.

The mutations were located at the catalytic site loop regions. The methionine at position 549 (L26) is unique in friedelin synthases from *M. ilicifolia*, *P. davidiana* and *T. wilfordii*. The phenylalanine at position 473 (L23) was changed to a tryptophan residue due to the possible interaction between residues Phe473 and Trp612, as seen in the homology-modeled enzyme structure (Supplementary Figure S2), whereas leucine 552 (also in L26) was changed to Phe because of the way friedelin fits into the friedelin synthase active site.

As shown in Figure 3, loop L26 is the entrance of the catalytic site, while loops L18 and L23 belong to the same cavity, on opposite extremities. Conformational freedom is assured exclusively to L26, which is oriented towards the solvent direction. L18 and L23 block the helix inside the catalytic site, affecting the movement of this region.

Wild type and mutants Phe473Trp, Met549Ser and Leu552Phe of *Mi*FRS were prepared and heterologously expressed in *S. cerevisiae* with decreased expression of *ERG7*, coding for the constitutive lanosterol synthase that competes for the precursor 2,3-oxidosqualene. Gas chromatography coupled to mass spectrometry detection was used to check the friedelin production level and the presence of other triterpenes in *S. cerevisiae* extracts. Changes in triterpene production were evaluated according to changes in the enzyme structure. Possible changes in amino acid interactions with different residues, intermediate carbocations or active site were analyzed by the enzyme model and docking.

#### 2.1.1. Phe473 Is Essential for Friedelin Activity

The residue 473 was mutated due to its conservation, its position in the catalytic cavity and its possible interaction with Trp612 by forming a sandwich with B-ring of the triterpene (Supplementary Figure S2). We observed that the presence of an adequate residue was important to the whole enzyme activity, since no friedelin or any other triterpene production was observed. The mutant MiFRS^Phe473Trp^ led to the loss of enzyme activity with no triterpene production.

Phenylalanine and tryptophan are nonpolar aromatic amino acids. The difference between them is an extra pyrrole group on the tryptophan chain. The increase in carbon on the side chain might influence the accommodation of the 2,3-oxidosqualene substrate in the friedelin synthase active site, inhibiting the cyclization reaction and friedelin and/or the production of other triterpenes, as shown in Supplementary Figure S3. In contrast, the corresponding mutant Phe474Trp in β-amyrin synthase from *Medicago tirucalli* (EtAS) exhibited similar activity as wild-type EtAS, producing β-amyrin and a bicyclic triterpene [25].

**Figure 4:**
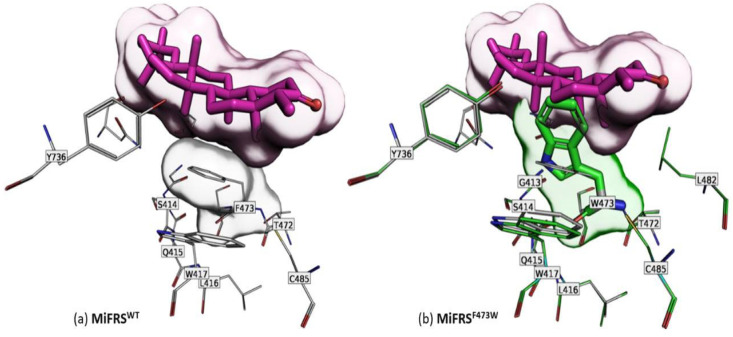
Secondary alignment (or side-by-side representation) of wild protein (*Mi*FRS) (gray-stick carbons) with the *Mi*FRS^Phe473Trp^ mutant (green-stick carbons). The surface highlights the volume adopted by the friedelin molecule in the catalytic cavity. The green stick indicates the mutated residue. (**a**) Favorable interaction between the wild-type Phe473 residue and substrate. (**b**) Steric bulk of Trp473 residue at the active site of MiFRS^Phe473Trp^. Interaction between Trp417 and Trp473 residues is favorable, which blocks the substrate entrance inside the pocket.

Docking friedelin in the MiFRS^Phe473Trp^ model showed that the replacement of phenylalanine with tryptophan increased the volume occupied inside the active site, compared to the apoenzyme form (Figure 4). When the substrate enters, there was an apparent steric blockage due to the indole group of the Trp473 side chain. The conformation adopted in the model is the only possible among all tested because the side chains of residues Cys485, Trp417, Ser411 or Leu482 block the possible rotamers. In addition, when the substrate was absent, the mutant indole ring (Trp473) interacts through π-electron conjugation with Trp417, filling the catalytic space and not reaching the ideal conformation to interact favourably with the substrate. By contrast, because Phe473 is a less bulky residue, there was no mobility problem with this residue in accommodating the substrate.

The activity of the EtAS^Phe474Trp^ mutant suggested that decreased specific product (β-amyrin) production and deficient bicyclic triterpene formation were because of a conformational change due to the larger tryptophan residue inside the catalytic cavity changing the B-ring formation [25]. This study also observed that other amino acid mutations led to different production of triterpenes or amounts of β-amyrin produced but did not interrupt triterpene formation.

However, MiFRS^Phe473Trp^ interrupted the formation of any triterpene because of the residue mobility blockage by residues around and interaction with Trp417, which obstructed the substrate interaction (Figure 4a). In EtAS^Phe474Trp^, there is a cation-π interaction stabilizing the intermediate, and the authors reported that Trp residue changed the active site conformation because of its larger volume. However, it was not reported as a blockage of the site, as analyzed in mutant MiFRS^Phe473Trp^.

We agree that the Phe473 residue is essential to the enzyme activity due to the steric hindrance in the catalytic cavity, and Phe473 possibly interacts by π-electrons, stabilizing the substrate.

#### 2.1.2. Met549 and Leu552 Influence the Specificity of MiFRS

The mutation *Mi*FRS^Met549Ser^ was analyzed because the methionine residue at this position is only conserved among friedelin synthases, except for *Kd*FRS. The human lanosterol synthase, has a serine as residue, but in other tetracyclic triterpenes, a small hydrophobic valine or alanine residue is conserved, and the majority of pentacyclic triterpene synthases hold a small polar threonine at this position (Supplementary Figure S1).

The other mutation analyzed, Leu552Phe, was selected because most of the OSC enzymes bear phenylalanine in the 552 position, including *Kd*FRS, while the friedelin synthases from *M. ilicifolia*, *P. davidiana* and *T. wilfordii* have a leucine residue in this position (Supplementary Figure S1). Therefore, Met549 and Leu552, both in L26 towards the active site, can be related to *Mi*FRS specificity.

**Figure 5:**
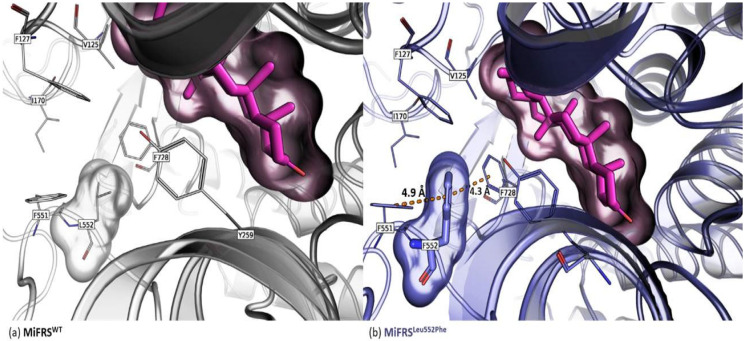
Secondary alignment (or side-by-side representation) of wild protein (*Mi*FRS) (gray-lines carbons) with the *Mi*FRS^Leu552Phe^ mutant (blue-lines carbons). The surface highlights the volume adopted by the friedelin molecule in the catalytic cavity. The blue stick residue indicates the mutated residue. (**a**) The mutation approached the Phe728 (not shown) residue of catalytic site, with a π-electron type interaction that stabilizes the C-ring, decreasing the enzyme specificity. (**b**) Another possibility of rotamer is impaired due to the collapse of other main or side chain residues.

Both mutants *Mi*FRS^Met549Ser^ and *Mi*FRS^Leu552Phe^ yield friedelin, α-amyrin and β-amyrin as products (Supplementary Figure S3). Friedelin production remained the same by the *Mi*FRS^Leu552Phe^ mutant compared to friedelin production by the wild-type enzyme. This can be explained by a new interaction, not present in the wild-type enzyme, observed in molecular modelling and docking studies. Residue Phe552 was sandwiched with two other phenylalanine residues (551 and 728). The replacement of leucine with phenylalanine (Leu552Phe) approached the Phe728 residue interacting through π-electron. Thus, when Phe728 was outside of the catalytic environment, the expansion of carbocation intermediates beyond the C-ring (beyond the oleanyl cation) was decreased, decreasing the enzyme specificity (Figure 5a). Different rotamer possibilities were hindered by clashing of the main or side chains of other residues, but there was a chance that 6.6% of Phe552 faced towards the active site and altered the conformation of Phe728 towards the substrate, impairing its ligation to the active site (Figure 5b).

**Figure 6:**
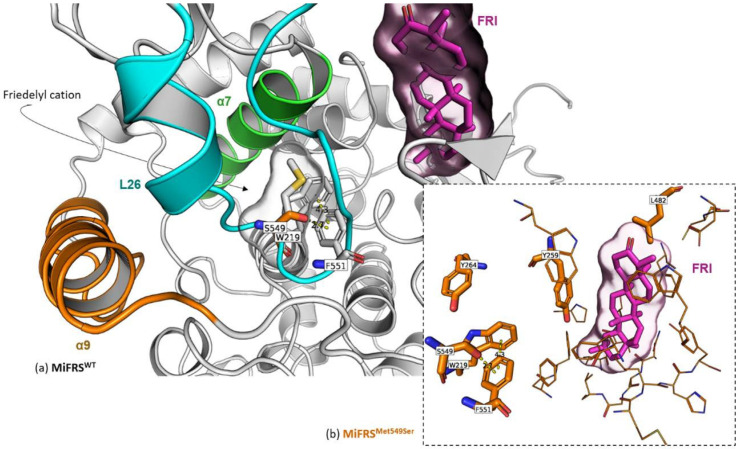
Secondary alignment (or side-by-side representation) of the wild protein (*Mi*FRS) (grey stick carbons) with *Mi*FRS^Met549Ser^ mutant (orange stick carbons). The surface highlights the volume adopted by the friedelin molecule in the catalytic cavity. Colored cartoons represent important protein regions: i. membrane contact α9 (orange), ii. barrel center entrance, helix α7 (green) and iii. catalytic loop L26. (**a**) The native Met549 occupies the route for the substrate achieves the catalytic. On the other hand, the single mutation Met549Ser stabilizes aromatic residues Tyr264 and Trp219; anion-π interaction between hydroxyl of Ser549 residue and the Phe551 residue is created. Interactions between the Tyr264 and Trp219 residues and the anion at the Ser549 mutated residue keep the site opened near ring C. (**b**) Stabilization of the Ser549/Phe551/Trp219/Tyr264 complex. The anion-π interaction is distanced by 2.7 Å, whereas the T-shaped π-π interaction is 4.3 Å closed.

Sandwich stabilization within the enzyme residues or residue-intermediate can lead to changes in the enzyme product. Wild-type SAD1 (from *A. strigosa*) produces β-amyrin, but the mutant Ser728Phe created a sandwich with residue Phe725, and the carbocation of the dammarenyl cation gave rise to a tetracyclic triterpene because the carbocation expansion was compromised [20]. Here, the oleanyl cation stabilized the mutant Phe552/Phe551/Phe728 sandwich, which was a slightly remote from the active site, simultaneously allowing the formation of β-amyrin and α-amyrin and carbocation expansion to a small proportion of friedelyl cations, followed by friedelin production. In contrast, the friedelin production of the *Mi*FRS^Met549Ser^ mutant was increased by two-fold when compared to the wild-type *Mi*FRS. In that case, the aromatic residues Tyr264 and Trp219 are stabilized by the serine from an anion-π interaction between the hydroxyl and Phe551 residue, closing the complex [26]. The native structure showed an unfavorable angle of 98° from the interaction of Met549 with Phe551. Moreover, the side chain of Met549 in the structural centre retarded substrate movement to achieve the catalytic pocket. When serine was at position 549, the Ser549/Phe551/Trp219/Tyr264 complex stabilizes, preventing the movement of the L26 loop and keeping the active site entrance open for substrate binding and product exit. Consequently, this allowed the interaction of Tyr259 and Leu482 in enzymatic catalysis, which explains the increase in triterpene production (Figure 6). In our study, the aromatic groups in Tyr264 and Trp219 created a sandwich with the anion in the Ser549 mutated residue close to the C-ring, making it possible to stabilize the oleanyl cation and produce β- and α-amyrin, although at a small proportion compared to friedelin production (Figure 6).

#### 2.1.3. Met549Ser Is More Efficient in Friedelin Production

**Figure 7:**
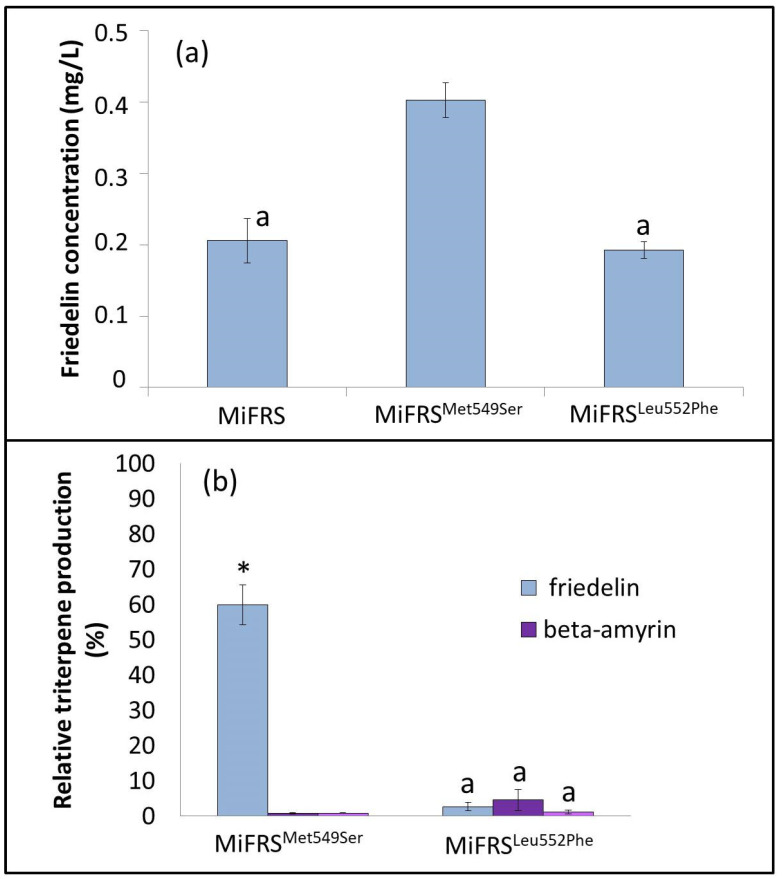
Concentration of friedelin in fractions extracted from *S. cerevisiae* yeast, expressing *Mi*FRS mutants. (**a**) Concentration of friedelin is expressed in mg/L of culture medium. (Complementary Data in Supplementary Material, Table S4). (**b**) Proportion between friedelin, β-amyrin and α-amyrin produced in the mutants of *Mi*FRS. A statistically significant difference is indicated by one asterisk (*) on the top of the bar, after analysis of variance. Bars with the same letter denote an absence of a statistically significant difference between each other on the quantification friedelin in the respective season (*p* > 0.05; one-way ANOVA with Tukey’s test).

Friedelin is a compound considered medicinally important due to the variety of biological activities attributed to it and is the main precursor of quinonemethide triterpenes with promising antitumor activity. In this sense, it is necessary to conduct more mutagenesis studies aimed at modifications in the friedelin synthase active site that make the enzyme more efficient and, thus, increase the production of friedelin. In our studies, the *Mi*FRS^Met549Ser^ mutant produced approximately two times more friedelin compared to the wild-type enzyme, with a culture medium concentration of 0.40 mg/L (Figure 7a). In addition, the *Mi*FRS^Met549Ser^ and *Mi*FRS^Leu552Phe^ mutants also produced β-amyrin and α-amyrin. The proportions between the heterologously produced triterpenes are described in Figure 7b. The exchange of amino acid residues is intended to increase the production of the substance of interest. However, it is important to emphasize that it is necessary not only to improve the catalytic activity but also to improve the enzyme specificity. The *Mi*FRS^Leu552Phe^ mutant lost its specificity and started to produce more β-amyrin than friedelin. The *Mi*FRS^Met549Ser^ mutant was more efficient from a quantitative point view, but it also lost its specificity and started to produce the other triterpenes, although in smaller quantities. Thus, it is still necessary to understand the active site of friedelin synthase and increase the production of friedelin, ensuring its specificity.

## 3. Discussion

Increased friedelin production by the friedelin synthase mutant was also observed in TwOSC1^Thr502Glu^. TwOSC1 is a friedelin synthase from *T. wilfordii* producing friedelin, β-amyrin and α-amyrin. The Thr502Glu mutant continued producing a similar amount of β-amyrin and α-amyrin but increased the production of friedelin by almost 45%, most likely by influencing the release of the triterpene [22]. The production of friedelin by *Mi*FRS^Met549Ser^ increased 96%, but *Mi*FRS also started producing β-amyrin and α-amyrin; although β-amyrin and α-amyrin were not observed with wild-type *Mi*FRS, they could be the product of diminished rearrangement due to the open entrance and exit, a result of the reduced mobility of L26. Regardless, this increase in production indicated an opportunity to improve the yield of friedelin in *S. cerevisiae*.

## 4. Materials and Methods

### 4.1. Site-Directed Mutagenesis Reaction

PCR to the replace the residues of interest was performed using 2 µL of plasmid [pSP_P*_TEF1_*-MiFRS-6xHis, P*_PGK1_*-t*HMG1*_*URA3*] as the DNA template and the Phusion^®^ High-Fidelity DNA Polymerase enzyme, according to the single primer amplification method [27]. The *Mi*FRS sequence (GenBank accession number MK526901) used for site-directed mutagenesis was codon optimized for further expression in *S. cerevisiae*. Obtained mutants were sequenced following the methods described elsewhere [19]. Forward and reverse primers for site-directed mutagenesis are described in Supplementary Table S3, and the primers used for sequencing are presented in Supplementary Table S4.

### 4.2. Heterologous Expression of MiFRS Mutants in S. cerevisiae

To evaluate possible changes in friedelin production by the formed mutants, the plasmids containing the friedelin synthase coding sequence and the obtained mutants were transformed individually in the *S. cerevisiae* VZL1434 strain background CEN.PK113-5D (*MATa MAL2-8c SUC2 ura3-52* P*_ERG7__∆_*::P*_KEX2_*) by the lithium acetate/polyethylene glycol method [28], and transformants were recuperated in synthetic complete medium without uracil (SC-U). Inocula and incubations of strains transformed with an empty plasmid or plasmids carrying wild-type *Mi*FRS or mutant sequences were performed separately in minimal medium as described previously [29].

### 4.3. Extraction of Biosynthesized Substances by the Heterologous System

The dried cell mass was weighed and transferred to borosilicate glass tubes (Pyrex, 16 × 100 mm) for further extraction with organic solvent. For this, the dried material was submitted to ultrasound extraction (Elmasonic S30H, ELMA) for 15 min (three extractions with 5 mL), using hexane solvent. All extractions were performed in triplicate.

### 4.4. Analysis of the Generated Products

Analytical curve samples and substances extracted from *S. cerevisiae* cells were analysed by gas chromatography coupled to mass spectrometry. Analytical curve was obtained with friedelin standard (Sigma-Aldrich, Sigma-Aldrich, St. Louis, MO, USA). The dried samples were resuspended in 200 μL of CHCl_3_. Cholesterol (Sigma-Aldrich) was used as an internal standard at a concentration of 50 μg/mL. Chromatographic analysis was performed on a gas chromatograph-mass spectrometer (SHIMADZU, QP2020C W/O RP230V) with an HP-5 column (30 m × 0.25 mm × 0.25 μm; Agilent Technologies) and the following conditions: injector temperature, 270 °C; heating ramp, 200 to 290 °C (10 °C/min); trap temperature, 200 °C for 3 min; interface temperature, 290 °C for 18 min; injection volume, 1 μL; split type injection mode, 1:10; carrier gas flow, 1.0 mL/min; total analysis time, 30 min; and mass/load ratio m/z, 35 to 600.

### 4.5. Protein Homology Modelling

The models were built following the methodology of homology modelling with the Modeler program [28,30]. The ViTaMIn interface was used as an aid in the development of models and mutations. The primary sequence of friedelin synthase from *Maytenus ilicifolia* (*Mi*FRS) was extracted from GenBank under code KX147270. The optimized sequence of *Mi*FRS for yeast expression is in GenBank accession number MK526901.The search for homologues resulted in 41% sequence identity with the enzyme oxidosqualene cyclase of *Homo sapiens*. The mutations were generated manually. After molecular modelling, the best models were selected based on the Ramachandran and DOPE energy diagrams. Alignments of secondary structures indicated high structural similarity (r.m.s.d < 0.2 Å).

### 4.6. Data Availability

The datasets analysed during the current study are available from the corresponding author on reasonable request.

## 5. Conclusions

Site-directed mutation studies were performed in vivo to enhance the understanding of the catalytic activity and specificity of friedelin synthase, in addition to evaluating the production of friedelin and other triterpenes by mutants. The mutation Phe473Trp led to the loss of enzymatic function, impacting friedelin formation. The yeast with mutations Met549Ser and Leu552Phe showed catalytic activity of friedelin synthase in conjunction with α-amyrin and β-amyrin production. In addition, the friedelin production in yeast with the mutation Met549Ser increased by two-fold compared with the wild-type gene. This result was explained by molecular modeling studies revealing that serine in the 549 position keeps the entrance of the active site open, allowing the substrate to bind and expelling the product, which increases the production of friedelin. Hence, it was also possible to conclude that the phenylalanine (Phe473) residue is crucial in maintaining the catalytic activity of friedelin synthase of *M. ilicifolia*, whereas the methionine (Met549) and leucine (Leu552) residues are important for the stabilization, rearrangement control and specificity of friedelin synthase.

## Supplementary Materials

The following are available online, Figure S1: Multiple global alignment in the wild MiFRS sequence. It is highlighted the mutated residues, Figure S2: Interaction between Phe473 residue and Trp612 residue, Figure S3: Chromatogram of substances used as standard: (a) friedelin, (b) β-amyrin and α-amyrin. Chromatogram of the hexane fractions of MiFRS (c), F473W (d), L552F (e) and M549S (f), Figure S4: Chromatogram of the hexane fractions of wild type of MiFRS: (a) squalene, (b) oxidosqualene, (c) cholesterol and (d) friedelin, Table S1: Sequences of the oxidosqualene cyclase enzymes used in multiple alignment with their accession number on GenBank, species and function, Table S2: List of forward and reverse primers for each site-directed mutation reaction, Table S3: List of forward primers used in sequencing for each site-directed mutation reaction, Table S4: Concentration of friedelin present in fractions extracted from S. cereviseae yeast, expressing MiFRS mutants.

## Sample Availability

Samples of friedelin compound, plasmids or strains are available from the authors. Please contact Correspondence author for more information.
