# Supplementary material for: The Methionine 549 and Leucine 552 Residues of Friedelin Synthase from Maytenus ilicifolia Are Important for Substrate Binding Specificity
Source: Molecules. 2021 Nov 11;26(22):6806. doi: 10.3390/molecules26226806 (PMC8617677; doi:10.3390/molecules26226806)

Molecules

The methionine 549 and leucine 552 residues of friedelin synthase from *Maytenus ilicifolia* are important for substrate binding specificity

Bruna F. Mazzeu[1], Tatiana M. Souza-Moreira[1], Andrew A. Oliveira[2], Melissa Remlinger[1], Lidiane G. Felippe[1], Sandro R. Valentini[3], Rafael V.C. Guido[2], Cleslei F. Zanelli[3] and Maysa Furlan[1]*

[1]*Instituto de Química, Univ. Estadual Paulista - UNESP,*

CP 355, 14800-900 Araraquara, SP, Brazil.

[2]*Centro de Pesquisa e Inovação em Biodiversidade e Fármacos, Instituto de Física de São Carlos, Universidade de São Paulo, São Carlos, SP 13563-120, Brazil.*

[3]*Faculdade de Ciências Farmacêuticas, Univ. Estadual Paulista-UNESP, Rod. Araraquara-Jaú km 1, Araraquara, SP 14801-902, Brazil*

*Corresponding author. Tel: +55-16-3301-9661; fax: +55-16-3301-9692.

E-mail address: maysa.furlan@unesp.br (M. Furlan)

Supplementary Material

Figure S1: Multiple global alignment in the wild MiFRS sequence. It is highlighted the mutated residues.

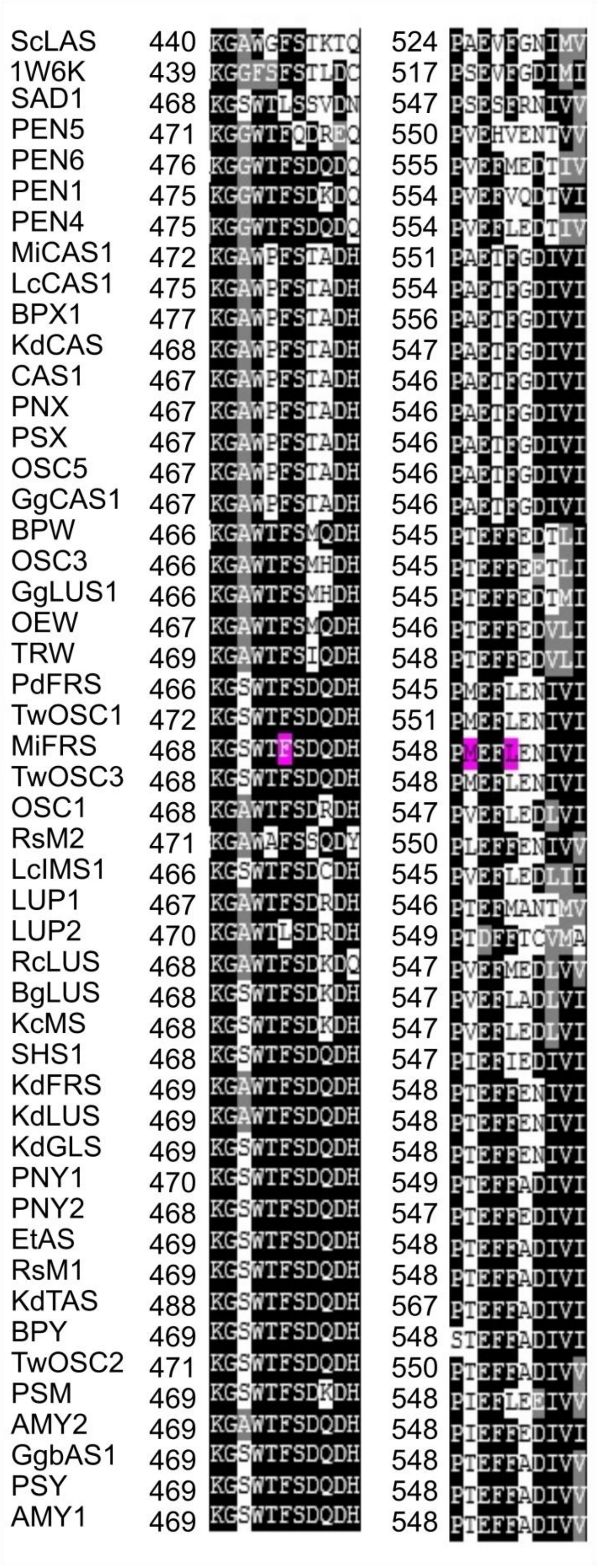

Table S1: Sequences of the oxidosqualene cyclase enzymes used in multiple alignment with their accession number on GenBank, species and function.

Accession number	Species	Function
AB257562.1	*Arabidopsis thaliana*	Arabidiol synthase (PEN1)
AY327541.1	*Arabidopsis thaliana*	Talianol synthase (PEN4)
BT020456.1	*Arabidopsis thaliana*	Marneral synthase (PEN5)
AB274959.1	*Arabidopsis thaliana*	Triterpene synthase multifuncional (PEN6)
NM_179572.1	*Arabidopsis thaliana*	Lupeol synthase 1 (LUP1)
NM_106545.3	*Arabidopsis thaliana*	Lupeol synthase 2 (LUP2)
NM_126681.2	*Arabidopsis thaliana*	Cycloartenol synthase (CAS1)
AB263204.1	*Rhizophora stylosa*	Triterpene synthase multifuncional (RsM2)
AB289586.1	*Bruguiera gymnorhiza*	Lupeol synthase (BgLUS)
AB257507.1	*Kandelia candel*	Triterpene synthase multifuncional (KcMS)
DQ268869.1	*Ricinus communis*	Lupeol synthase (RcLUS)
AB058643.1	*Luffa cylindrica*	Isomultiflorenol synthase (LcIMS1)
AB037203.1	*Glycyrrhiza glabra*	β-amyrin synthase (GgbAS1)
AB181244.1	*Lotus japonicus*	β- amyrin synthase (OSC1)
AB034802.1	*Pisum sativum*	β- amyrin synthase (PSY)
AF478453.1	*Medicago truncatula*	β-amyrin synthase (AMY1)
AF478455.1	*Lotus japonicus*	β-amyrin synthase multifuncional (AMY2)
AB034803.2	*Pisum sativum*	amyrin synthase mista (PSM)
AB009030.1	*Panax ginseng*	β-amyrin synthase (PNY1)
AB014057.1	*Panax ginseng*	β-amyrin synthase (PNY2)
AB055512.1	*Betula platyphylla*	β-amyrin synthase (BPY)
HM623868.1	*Kalanchoe daigremontiana*	Taraxerol synthase (KdTAS)
AB263203.1	*Rhizophora stylosa*	Triterpene synthase multifuncional (RsM1)
AB206469.1	*Medicago tirucalli*	β-amyrin synthase (EtAS)
HM623870.1	*Kalanchoe daigremontiana*	Friedelin synthase (KdFRS)
HM623869.1	*Kalanchoe daigremontiana*	Glutinol synthase (KdGLS)
HM623871.1	*Kalanchoe daigremontiana*	Lupeol synthase (KdLUS)
AB025343.1	*Olea europaea*	Lupeol synthase (OEW)
AB025345.1	*Taraxacum officinale*	Lupeol synthase (TRW)
AB181245.1	*Lotus japonicus*	Lupeol synthase (OSC3)
AB116228.1	*Glycyrrhiza glabra*	Lupeol synthase (GgLUS1)
AB055511.1	*Betula platyphylla*	Lupeol synthase (BPW)
AB181246.1	*Lotus japonicus*	Cycloartenol synthase (OSC5)
AB025968.1	*Glycyrrhiza glabra*	Cycloartenol synthase (GgCAS1)
AB009029.1	*Panax ginseng*	Cycloartenol synthase (PNX)
HM623872.1	*Kalanchoe daigremontiana*	Cycloartenol synthase (KdCAS)
AB055509.1	*Betula platyphylla*	Cycloartenol synthase (BPX1)
APG38073.1	*Maytenus ilicifolia*	Friedelin synthase (MiFRS)
AAA16975.1	*Saccharomyces cerevisiae*	Lanosterol synthase (ScLAS)
P48449.1	*Homo sapiens*	Lanosterol synthase (1W6K)
BAK52535.1	*Aster tataricus*	Shionone synthase (SHS1)
AJ311789.1	*Avena strigosa*	β-amyrin sintase (SAD1)

ART66198.1	*Populus davidiana*	Friedelin synthase (PdFRS)
AWK97810.1	*Tripterygium wilfordii*	Friedelin synthase (TwOSC1)
AWK97811.1	*Tripterygium wilfordii*	β-amyrin synthase (TwOSC2)
AWK97812.1	*Tripterygium wilfordii*	Friedelin synthase (TwOSC3)

Table S2 - List of forward and reverse primers for each site-directed mutation reaction.

Primer	**Sequence 5'-3'**	**Tm (° C)**
**F473Wf** (VZO2355)	CAAGGGTAGTTGGACATGGTCCGATCAAGACCACGG	67,1
**F473Wr** (VZO2356)	CCGTGGTCTTGATCGGACCATGTCCAACTACCCTTG	67,1
**M549Sf** (VZO2361)	GATGGAAATGTTGAACCCATCTGAATTCTTGGAAAACATCG	62,8
**M549Sr** (VZO2362)	CGATGTTTTCCAAGAATTCAGATGGGTTCAACATTTCCATC	62,8
**L552Fr** (VZO2364)	GATGACGATGTTTTCAAAGAATTCCATTGGG	58,5

The underlined region indicates the bases of the respective amino acid residue mutated. The letters *f* and *r* in the primers correspond to forward and reverse, respectively.

Table S3 - List of forward primers used in sequencing for each site-directed mutation reaction.

Primer	**Sequence 5'-3'**	**Verified mutations**
**369/484seq** (VZO2372)	GGGACTCTTTGTATGTTGCATC	F473W
**534/612seq** (VZO2373)	GTTGCTTGTTAGCTGCAACC	M549S, L552

Figure S2: Interaction between Phe473 residue and Trp612 residue.

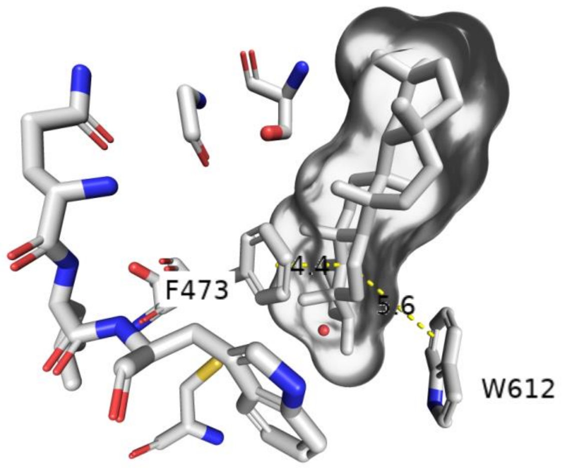

Table S4: Concentration of friedelin present in fractions extracted from *S. cereviseae* yeast, expressing *Mi*FRS mutants.

Sample	Friedelin peak area average t_R ~ 21,100 min	Cholesterol peak area average t_R ~ 14,706 min	Friedelin area/ Cholesterol area	[friedelin] (mg/L)*
*Mi*FRS – Hex	897656,7	3948327	0,227351	0,206±0,002
M549S - Hex	5548441	2112643	2,626303	0,403±0,002
L552F - Hex	268982,7	4824556	0,055753	0,192±0,001

* Concentration of friedelin in mg/L of culture medium.

Figure S3: Chromatogram of substances used as standard: (a) friedelin, (b) β-amyrin and α- amyrin. Chromatogram of the hexane fractions of *Mi*FRS (c), F473W (d), L552F (e) and M549S (f).

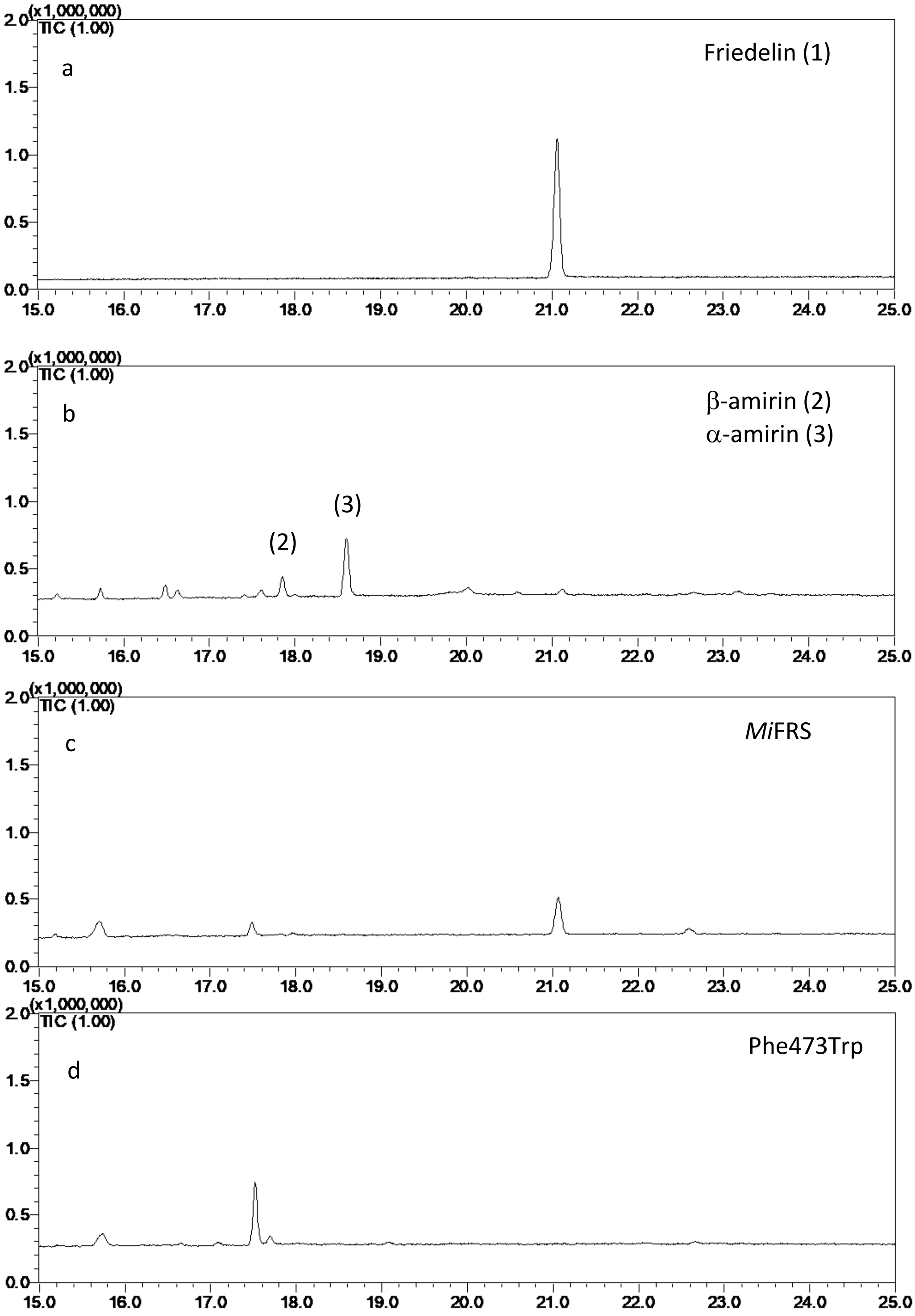

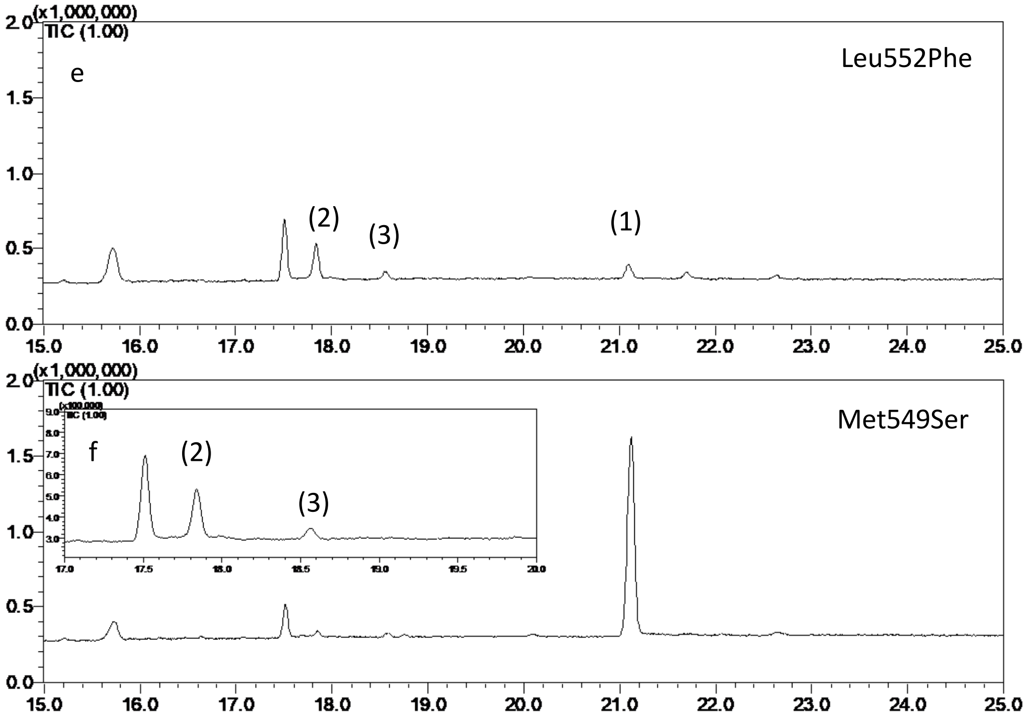

Figure S4: Chromatogram of the hexane fractions of wild type of MiFRS: (a) squalene, (b) oxidosqualene, (c) cholesterol and (d) friedelin.

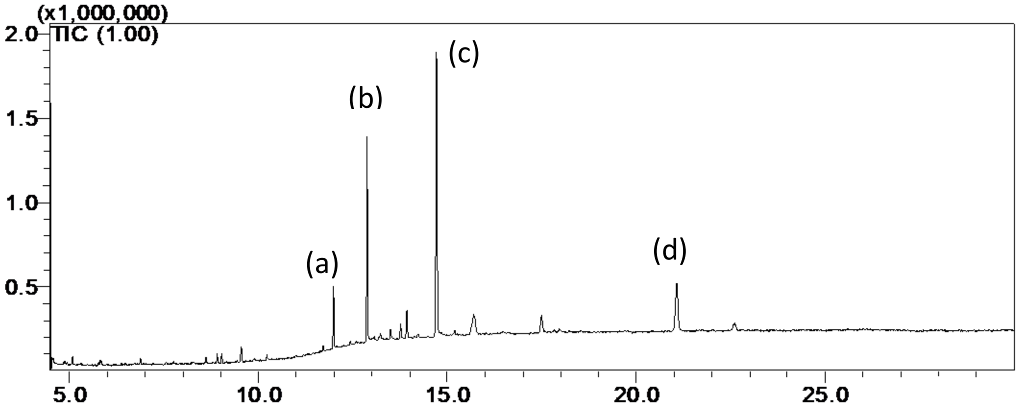

Supplement: Supplementary file 1 [file molecules-26-06806-s001.zip › molecules-1332656-supplementary.pdf]
